# Soft tissue repair for tibialis anterior tendon ruptures using plate and screw fixation technique in combination with anterolateral thigh flaps transplantation

**DOI:** 10.1186/s13018-015-0278-5

**Published:** 2015-09-17

**Authors:** Haijun Mao, Guanyue Xu

**Affiliations:** Department of Orthopedics, Drum Tower Hospital, Medical School of Nanjing University, Zhongshan Road 321, Nanjing, 210008 China

**Keywords:** Tibialis anterior tendon, Rupture, Tendon transfer, Plate and screw fixation technique, Anterolateral thigh flaps

## Abstract

**Background:**

Traumatic ruptures of the tibialis anterior tendon are rare but can cause substantial functional deficiencies. This study aimed to evaluate the feasibility of a surgery for soft tissue repair of traumatic rupture of the tibialis anterior tendon by using a plate and screw fixation repair in combination with the free anterolateral thigh flaps transplantation.

**Methods:**

Eight consecutive patients with anterior tibialis tendon ruptures who visited orthopedics departments from February 2008 to February 2012 were included in our study. The ruptured tendon was reconstructed with plate and screw fixation technique, and the tissue defects were repaired with anterolateral thigh free flaps. The complications and American Orthopedic Foot and Ankle Society (AOFAS) ankle-hindfoot scores were evaluated. Postoperative manual strength test was performed using a 0 to 5 scale.

**Results:**

All flaps survived without any complications. The average preoperative and postoperative AOFAS ankle-hindfoot scores of the patients were 51 and 95, respectively. Good ankle dorsiflexion strength against strong resistance was observed in eight ankles postoperatively (manual strength of one patient was 4/5, the others were 5/5), and a substantial improvement in strength was noted compared with the preoperative examination.

**Conclusion:**

Soft tissue repair for tibialis anterior tendon rupture using plate and screw fixation technique in combination with anterolateral thigh flaps transplantation is a feasible technique and yield satisfactory results.

## Highlights

1. A plate and screw fixation technique was applied for repair of tibialis anterior.
2. Anterolateral thigh flaps were used for the soft tissue reconstruction.
3. All flaps survived without any complications.
4. Patients showed satisfactory ankle-hindfoot scores and ankle dorsiflexion strength.

## Introduction

Either traumatic or atraumatic ruptures of the tibialis anterior tendon are uncommon. Traumatic rupture is usually associated with laceration or blunt trauma from injuries. It is caused by an acute trauma accompanied with osseous or soft-tissue injuries as well as pain and weakness in dorsiflexion of the ankle [1, 2]. Physical symptoms of traumatic ruptures include a pseudotumor presented at the anteromedial aspect of the ankle and loss of the normal contour of the tibialis anterior tendon [2]. Therefore, it is troublesome because the ruptures of tibialis anterior tendon related to patient’s daily life.

There are two treatment options for traumatic rupture: conservative treatment with foot orthosis and surgical treatment for tendon reconstruction [3]. Reconstruction of this tendon to restore ankle dorsiflexion and inversion includes end-to-end repair, tendon transfer, or allograft augmentation [4–6]. The rupture occurred with a 2- to 3-cm avascular lesion, and tissue defects makes a direct end-to-end suture impossible [7, 8]. Therefore, an island or free flap may be required for tendon transfer reconstruction. However, clear guidelines about the treatment of these injuries are currently unavailable due to the limited reports in orthopedic literatures.

The present study described a surgical technique by using plate and screw fixation with anterolateral thigh flap transplantation to reconstruct traumatic ruptures of the tibialis anterior tendon.

## Materials and methods

### Patients

Eight consecutive patients (male/female, 6/2) aged from 24 to 46 with a traumatic rupture of anterior tibialis tendon from February 2008 to February 2012 were included in our study. The ruptures (right/left, 5/3) from all these patients occurred because of direct blunt trauma of the tendon accompanied by osseous or soft tissue injuries.

Rupture of tibialis anterior tendon was diagnosed based on history and physical examination. All patients presented a chief complaint of functional deficiency, such as weak dorsiflexion of the foot, unsteady gait, limping, and increased fatigue during walk. On the physical examination of the patients’ pseudotumor at the anterior part of the ankle, weak dorsiflexion of the foot, abnormal contour of the tendon, and hyperextension of toes were determined. Besides, magnetic resonance imaging was performed for assessing the retraction of the tendon and confirming the diagnosis.

All patients signed the informed consent and were available to be followed up. In our study, the retrospective review was approved by the Ethics Committee of Drum Tower Hospital and were conducted based on medical records, physical examinations, and final patient interviews.

### Surgical technique

Surgeries were performed in the affiliated Drum Tower Hospital of Nanjing University Medical School, China. After general anesthesia, patients were treated with a small incision above the superior extensor retinaculum of their legs. The extensor retinaculum was left intact if possible to avoid the adhesion of the tendons.

Debridement was performed for the proximal and distal parts of the tendon. If the tendon could be connected together directly, a direct tendon repair was carried out. If not, an interpositional tendon graft, such as plantaris tendon (two patients), extensor digitorum longus tendon (one patient), and peroneus tertius tendon (one patient), was inserted to bridge the gap and reinforce the repair. Then, the tibialis anterior tendon was fixed to the anatomical insertions with plate and screw fixation technique.

Direct tendon repairs were performed in the other four cases. The end of the tibialis anterior tendon was placed on its anatomical insertion, and then, a mini-plate was pressed on it and screwed. The diameter of the harvested grafts was smaller than the tibialis anterior. In most patients, this tendon could be doubled, which usually resulted in a graft with a diameter of 5 to 6 mm. The ankle and foot were held in maximal dorsiflexion and maximal supination, respectively, to determine the final length of the tendon graft. One end of the grafted tendon was fixed using plate and screw fixation technique.

When the bony attachment was performed, the tendons were sutured to each other with Vicryl suture side to side. Finally, the tissue defect was repaired with free anterolateral thigh flaps. In this study, the anterolateral thigh flaps was used in all cases.

### Postoperative care

After surgery, patients were treated with short-leg cast immobilization for the first 2 weeks to maintain the ankle in 0° of dorsiflexion. Weight bearing in the cast was allowed in the succeeding 2 weeks. The duration of cast immobilization was partly dependent on surgeon’s perception of repair quality during surgery. At 6 weeks postoperatively, weight bearing and full dorsiflexion were allowed. Plantar flexion was gradually increased.

### Evaluation of postoperative manual strength and AOFAS scores

The postoperative manual strength test was conducted by using a 0 to 5 scale [2, 9]. In this scale system, 0 means no evidence of joint motion or muscle contraction, 1 (trace) means evidence of muscle contraction but no joint motion, 2 (poor) means a certain motion range limited by gravity, 3 (fair) means a motion range against gravity, 4 (good) means a motion range against a slight resistance, and 5 (normal) means a motion range against strong resistance. The American Orthopedic Foot and Ankle Society (AOFAS) ankle-hindfoot score was used for preoperative and postoperative evaluation [10]. This 100-point standard rating system is designed to compare the results of different treatment methods in patients with the same disorder. The anti-hindfoot AOFAS scores evaluate pain (50 points), function (including gait, range of motion, and strength) (40 points), and alignment (10 points).

## Results

### Patients’ characteristics

The average age of eight atraumatic rupture patients was 32 years (range, 24–46 years). Among these patients, five were injured by vehicle accident, two were injured by heavy subjects, and one was injured during machine operation. A direct tendon repair was carried out in five patients, and the others were suffered from tendon transplant (Table 1).

**Table 1:** Basic characteristics of patients included in our study

Patients no.	Gender	Ages (year)	Causes	Operation methods	Transplant tendons	Following up time (month)	Preoperative AOFAS	Postoperative AOFAS	Postoperative strength test	Complications
1	Female	24	Vehicle accident	Direct	No	12	46	96	5	Pain
2	Female	26	Vehicle accident	Direct	No	13	43	96	5	No
3	Male	28	Injured by heavy subjects	Indirect	Plantaris tendon	12	51	90	4	No
4	Male	32	Vehicle accident	Indirect	Peroneus tertius tendon	11	55	96	5	No
5	Male	46	Vehicle accident	Direct	No	14	53	95	5	No
6	Male	30	Machine injury	Indirect	Extensor digitorum longus tendon	12	57	94	5	No
7	Male	27	Injured by heavy subjects	Direct	No	11	56	98	5	No
8	Male	43	Vehicle accident	Indirect	Plantaris tendon	12	47	95	5	No

### Postoperative manual strength

Good ankle dorsiflexion strength against strong resistance was observed in eight ankles postoperatively (Table 1). A substantial improvement in strength was noted compared with the preoperative examination. All patients were able to walk without a visible limp. However, one of them had a little residual weakness in dorsiflexion compared with the uninjured side, though physical examination showed he had 5/5 strength (at 12-month follow-up). Another patient with 4/5 strength had a little claudication and hyperextension of the toes when walking at 12 months after surgery (statistical compared results were not shown because of the small sample size in our experiment).

### AOFAS ankle-hindfoot scores

All anterolateral thigh flaps survived without any complications, such as necrosis and infection, except for bloated appearance. The average preoperative and postoperative AOFAS ankle-hindfoot scores of the patients were 51 and 95, respectively. Active dorsiflexion was possible after 2 to 3 weeks.

### Complications

Complications were found in one of the eight patients at 12-month follow-up. She developed a regional pain syndrome. Based on comprehensive assessment, we thought that this complication may be caused by conglutination of the intermediate branch of the superficial peroneal nerve. After the related operation of releasing nerve, the patient did not feel residual pain and got back to normal gait. High levels of satisfaction with postoperative care and final outcome were obtained from all these patients.

### Case report

**Fig. 1:**
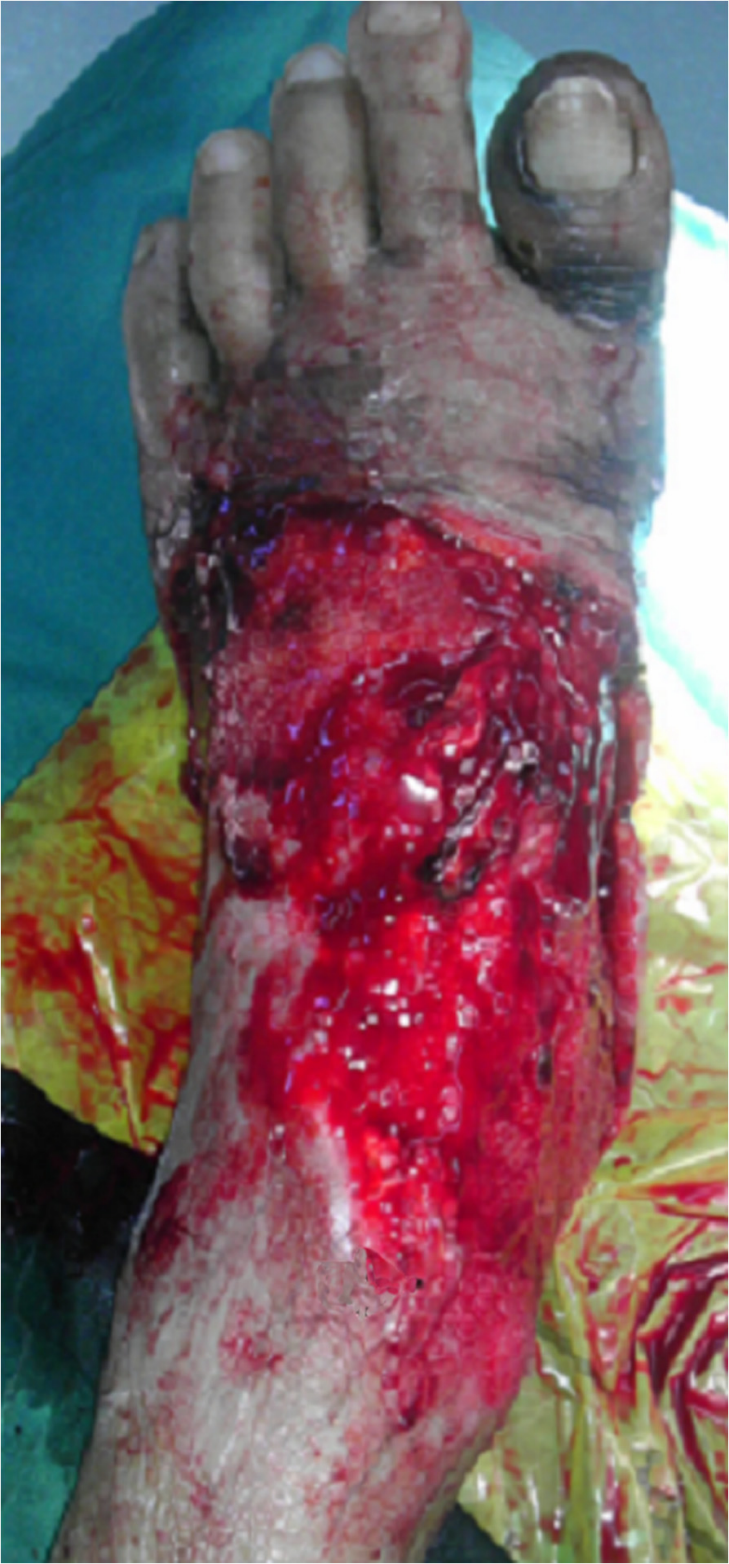
Preoperative view of the injured foot

**Fig. 2:**
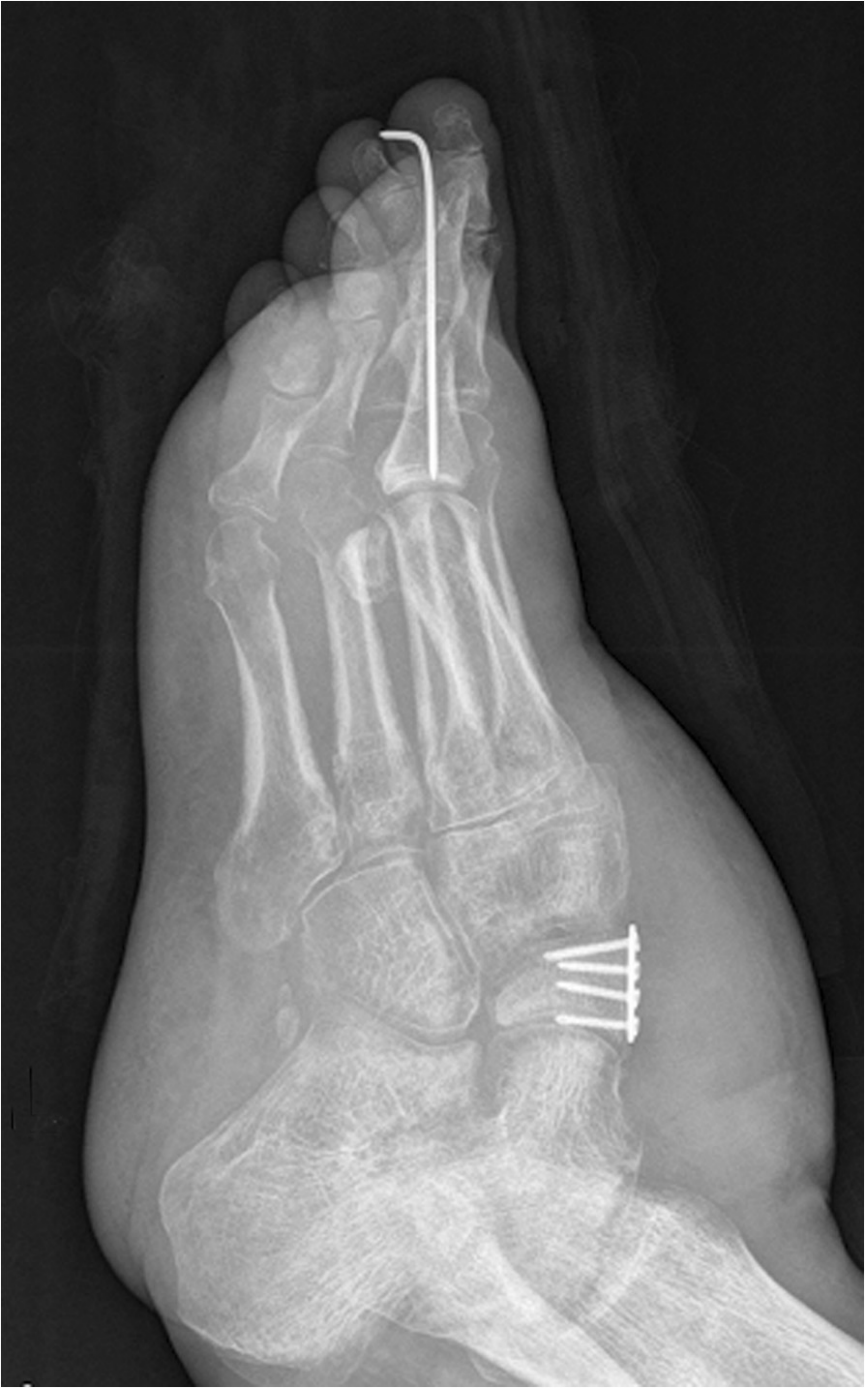
The tendon reconstruction by using the plate and screw technique

**Fig. 3:**
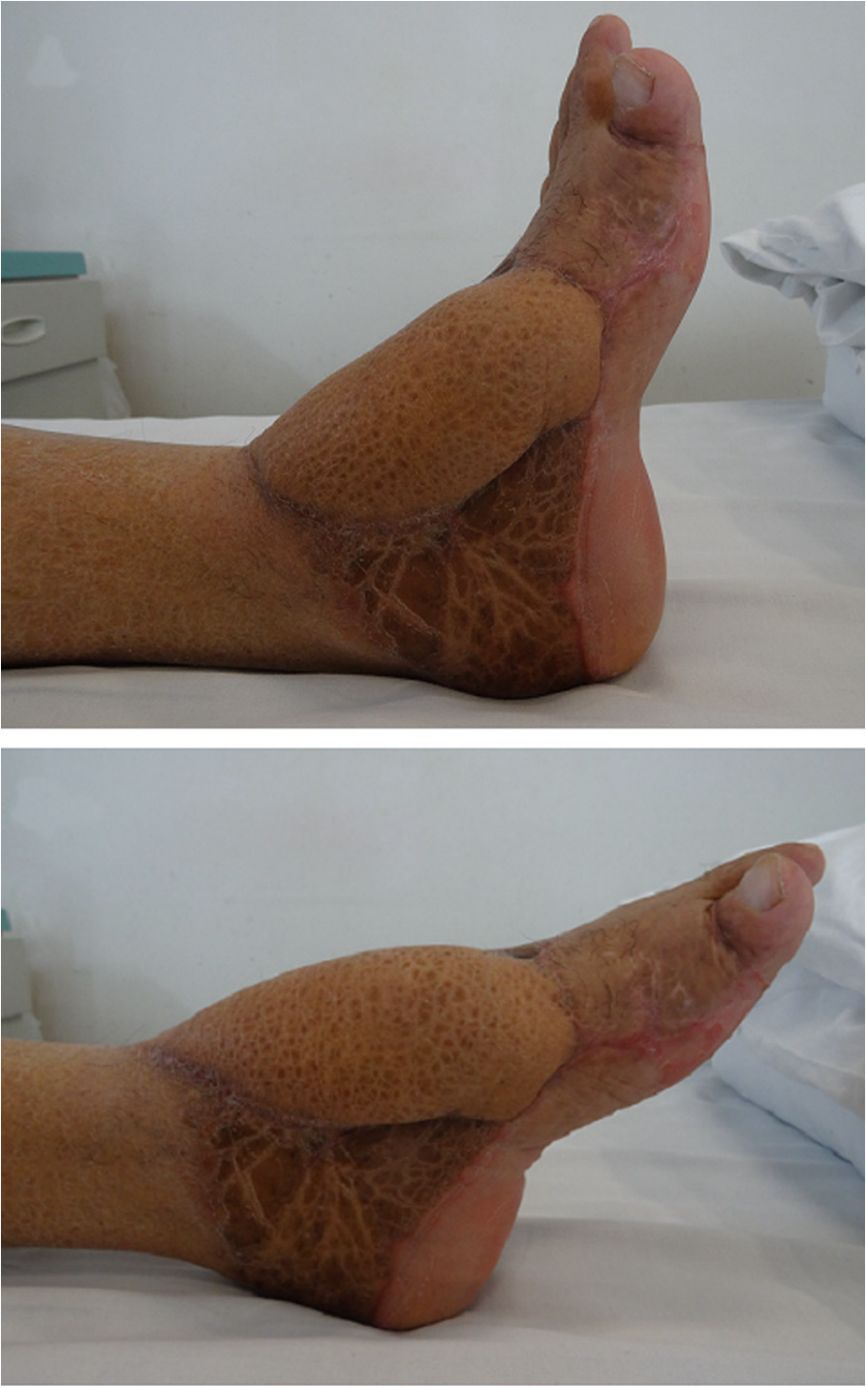
Postoperative view at 14 months

A 46-year-old man who suffered from a tissue defect and rupture of tibialis anterior tendon from a motor vehicle accident was reported here (Fig. 1). His preoperative AOFAS ankle-hindfoot score and ankle dorsiflexion strength were 53 and 3/5, respectively. One week later, a complete debridement was performed. We used the plate and screw technique to reconstruct the tendon insertion without tendon grafting (Fig. 2). The defect was reconstructed with anterolateral thigh free flap. After the operation, a short-leg cast was performed. Two weeks later, the flap was stable and the wound healed well. At a 14-month follow-up, both the postoperative AOFAS ankle-hindfoot score (95) and the ankle dorsiflexion strength (5/5) were much more elevated. Furthermore, he was able to walk without a visible limp (Fig. 3).

## Discussion

Reports about the tibialis anterior tendon ruptures are rare but can lead to considerable functional deficiencies. Most studies of tibialis anterior tendon rupture treatment have included isolated case reports and different treatment techniques [1, 7, 11–13]. In this study, we evaluated the plate and screw fixation with anterolateral thigh flaps for treatment of a group of patients with the tibialis anterior tendon ruptures. As a result, this technique is safe and efficient for traumatic rupture treatment.

Atraumatic ruptures and traumatic ruptures are easy to distinguish because of the different clinical presentations. The former occurs in low demand old patients, and often misdiagnosed in treatment, while the latter will happened in high demand younger patients with more disabilities. Markarian et al. [5] performed an operative (reconstruct the tendon with an adjacent tendon transfer) and a non-operative treatment (non-anatomic repair) on patients with anterior tibialis tendon ruptures and suggested that young active patients are advised to be treated with surgery while older patients should be treated conservatively in their series. However, Sammarco et al. [2] compared the outcomes of early surgical treatment and delayed surgical treatment (the treatment consisted of tendon graft and direct repair) and concluded that the desired level of activity of patients and the functional deficit, other than the age and the time from the injury alone, should be used for indicators of surgical repair. In our study, the subjects were aged from 24 to 46 years (mean, 32 years) and all of them expressed cure desires as soon as possible. Therefore, non-conservative surgery was performed for them.

Surgery techniques are important for cure of this disease. Interpositional autografts used plantaris tendon, extensor digitorum longus, extensor hallucis brevis, and achilles tendon for tendon graft is popular in recent literatures [2, 14]. In the present study, we chose anterolateral thigh flaps for the soft tissue reconstruction in these patients. As we know, the advantages of anterolateral thigh flap includes consistent and reliable anatomy, long pedicle, being far from the ablative site, and allowing two-team approach [15, 16]. Besides, it is feasible to create multiple skin paddles by recruiting additional perforators and reconstruct composite defect with chimeric flap by recruiting different tissue types based on a single pedicle [17]. It is also reported to have a low morbidity in donor site [18]. When a muscle component is required, the musculocutaneous anterolateral thigh flap is preferred, for the reason that it may decrease the operative time [19].

A suture anchor or a bio-tenodesis screw is often used for the reconstruction of the insertion of the tibialis anterior tendon. However, this study employed the plate and screw fixation technique to reconstruct the insertion because of its advantages. The screw can fix the tendon to the bone as point, and the plate can fix the tendon as flat. According to the physics formula of pressure (*p*) = force (*f*)/square (*s*), large square results in small pressure when muscle tension is constant. The plate and screw fixation technique may increase the square and decrease the pressure, thereby improve the firmness between the bone and the tendon. In this study, two patients removed the cast a week after the operation. At the last interview, both of them had a manual strength of 5/5 and walked without any visible limp. These results indicate that the technique decreased the duration of cast immobilization compared with previously published reports. However, no valid statistical approach could be performed because of the small sample size. Therefore, in our future research, we will use a large sample size to validate the results statistically.

During our operation, we tried different directions (perpendicular, parallel, or other angles) of the plate to the tendon and the direction of the muscular contraction. As a result, we found that placing the plate perpendicular to the tendon is relatively easy. We speculated that a share force exists between the plate and tendon if the plate is parallel to the tendon. In addition, a cutting action may be produced on the tendon after a long time of parallel.

There are several limitations included in this study. Firstly, the sample size is small because of the rareness of this disease. Secondly, it is retrospective and lacks a control group with non-operatively managed patients. Only younger patients who are symptomatic were included in our study. Therefore, the results may not represent all patients with tibialis anterior rupture. In addition, the AOFAS ankle-hindfoot score is not a validated instrument used as the clinical outcome measurement. Nevertheless, this study allows comparison of results because AOFAS ankle-hindfoot score is also used in other published studies [9].

In conclusion, we recommend surgical reconstruction of the traumatic ruptured tibialis anterior tendon using a plate and screw fixation technique for repair and an anterolateral thigh flap for soft tissue reconstruction. This technique allows early mobilization and yields satisfactory results.

## Abbreviations

AOFAS: American Orthopedic Foot and Ankle Society
